# Efficacy of nutrition education for the increase of symbiotic intake on nutritional and metabolic status in schizophrenic spectrum disorders: A two-arm protocol

**DOI:** 10.3389/fnut.2022.912783

**Published:** 2022-08-10

**Authors:** Alfonso Sevillano-Jiménez, Guillermo Molina-Recio, Juan Antonio García-Mellado, María García-Rodríguez, Rafael Molina-Luque, Manuel Romero-Saldaña

**Affiliations:** ^1^Córdoba-South Community Mental Health Unit, UCM Mental Health, Reina Sofia University Hospital, Córdoba, Spain; ^2^Department of Nursing, Pharmacology and Physiotherapy, University of Córdoba, Córdoba, Spain; ^3^Lifestyles, Innovation and Health (GA-16), Maimonides Biomedical Research Institute of Cordoba (IMIBIC), Córdoba, Spain; ^4^Psychiatry Service, Zamora Provincial Hospital, Zamora Welfare Complex, Zamora, Spain; ^5^Department of Nursing and Nutrition, Biomedicine sciences and Health Faculty, European University, Madrid, Spain

**Keywords:** prebiotic, probiotic, schizophrenia spectrum and other psychotic disorders, diet therapy, mental health

## Abstract

**Background/Objectives:**

The microbiota plays a vital role in the two-way communication between the gastrointestinal tract and numerous neuropsychiatric disorders, such as schizophrenia. Besides, the microbiota modulation through the use of psychobiotics (prebiotics and probiotics with nutraceutical action) is related to the improvement of the physical and psychopathological health. The objective to this study was to test the efficacy of prebiotic/probiotic dietary modulation in patients diagnosed with schizophrenia, attending to the nutritional and cardio-metabolic impact.

**Methods:**

Two-arms, double-blind, randomized in balanced blocks clinical trial of 6 months of intervention, will be developed in a group of 50 individuals. The control group will receive conventional dietary advice individually from specialized mental health nurses. In the intervention group, an individual dietetic-nutritional education program with high prebiotic and probiotic content (dairy and fermented foods, green leafy vegetables, high-fiber fruit, whole grains, etc.) will be developed by these nurses. Data will be collected on the psychopathological state, and blood test (at the beginning, at 3 and 6 months). The estimation of intestinal microbiota and the usual nutritional pattern will also be assessed at the beginning and 6 months, using a stool test. To evaluate the degree of adherence, the intervention group will fill a specific weekly record of the main dishes/food consumed. Anthropometric parameters will also be analyzed monthly.

**Discussion:**

The study is anticipated to establish feasibility an adequate dietary modulation with a high simbiotic content, leads to a significant improvement in the nutritional status and cardio-metabolic. Furthermore, it is presumed to reach a degree of evidence that allows establishing nutritional management as an effective therapeutic intervention in the psychopathological treatment of patients with schizophrenia spectrum disorders.

**Clinical Trial Registration:**

[www.ClinicalTrials.gov], identifier [NCT04366401].

## Background

Schizophrenia is a chronic mental illness characterized by significant clinical heterogeneity and a long evolution over time, determined by periods of psychotic exacerbation and phases of stabilization (1–3). The semiology of this nosological entity is established in positive and negative symptoms, with variable levels of dysfunction and clinical presentation, and having an essential impact on the patient’s quality of life (2, 4). Similarly, surrounding the schizophrenic spectrum, the existence of the associated neurocognitive impairment stands out, prevailing the disorders of social and occupational functioning, as well as a significant degree of disorganization (2, 3, 5, 6).

Many theories have tried to elucidate the origin of schizophrenia, where the complexity of its etiopathogenesis is a determining factor in establishing an appropriate, specific, and effective therapeutic approach (1, 7). In this sense, despite the numerous etiological premises, the glutamatergic (glutamate/GABA) and dopaminergic hypotheses have acquired greater strength in the development of schizophrenia (8, 9). In addition, however, recent studies have highlighted the theory of vagus nerve dysregulation as a possible etiological factor in the origin and aggravation of mental disorders (1, 5, 10), with numerous associated pathogenic mechanisms, particularly low-grade systemic inflammation and oxidative stress (7, 10, 11).

Undoubtedly, the traditional therapeutic approach has perceived the role of nutrition as a minor intervention in psychiatry, especially in psychotic disorders such as schizophrenia (12). However, the advances established in the last decade, mainly associated with the development of the holobionte theory and the evolution of metagenomics (11, 13), as well as the presence of new dietary patterns of low nutritional quality in different western societies (1, 3), have contributed significantly to the global understanding of the role of nutritional patterns on the functioning of the Central Nervous System (CNS), as well as on the possible mechanisms or etiological pathways of psychiatric disorders (1, 11, 12, 14).

In this regard, it is necessary to highlight the role of the intestinal microbiota (IM) and the intimate relationship it exerts on the numerous functions of the body, such as the development and maturation of the CNS, nutrition, immune response or systemic inflammation (7, 11, 12, 15). This effect is carried out through various established communication pathways: vagal nerve (primary), intestinal hormones, cytokines, exosomes, and microRNAs (10, 14–16). Thus, the existence of possible modifications in the concentration of this biota (considering average concentrations around 1,013 CFU/g) (17, 18), may trigger homeostatic alterations or aggravate pathogenic conditions, a fact commonly called dysbiosis (11–13, 17, 19). This concentration of microbiota is fundamentally determined by dietary patterns, genetic factors, iatrogenic antibiotherapy [highlighting the broad-spectrum ones, reducing the potential for small intestinal bacterial overgrowth (SIBO)], type of breastfeeding (maternal or formula), age, exercise, and continuous stress, among others (10, 18–20).

As a consequence of these discoveries, the concept of the “*Microbiota-Intestine-Brain Axis”* emerges. This term refers to the two-way communication pathway established between the CNS, the gastrointestinal tract, and the IM (1, 13–15, 18), mediated by the microbial metabolites of dietary products such as dietary fiber, tryptophan or arginine, as well as by endocrine and neuronal mechanisms (19, 21). The close relationship established between IM and the CNS lies in the production of a multitude of neurotransmitters essential for normal neuronal functioning, such as serotonin, GABA, dopamine or noradrenaline, among others (11–13, 19, 22). Similarly, IM exerts essential trophic, metabolic, and protective functions, which are a determining factor in the normal neuropsychiatric function (17, 22).

Thus, according to the theory of low-grade systemic inflammation, when a state of dysbiosis occurs in the symbiote IM, it generates a cascade of pro-inflammatory agents, such as lipopolysaccharide (10, 11, 23), a bacterial endotoxin, capable of modifying both the integrity and the permeability of enterocytes (12). This alteration triggers the release of pro-inflammatory cytokines [tumor necrosis factor ′α (TNF-′α) or interleukins type 6 or 1β (IL-6, IL-1β) (7, 11), both capable of altering intestinal tissue integrity], originating synergies between inflammation, increased oxidative stress and imbalance of energetic homeostasis. This cascade of reactions causes an increase in neurodegeneration and excitotoxicity, mediated by the vagus nerve (7, 12, 15). Thus, it has been shown that the activation of a state of low inflammation is related to a worse prognosis of schizophrenia concerning positive and negative symptoms, cognitive performance, and loss of brain volume (7, 21, 24). Similarly, alterations in specific pro-inflammatory cytokines or state markers have been described, especially in psychotic relapses or prodromal phases (IL-6, TGF-β, among others) (7, 10), as well as a decrease in their concentration after the introduction of antipsychotic treatment, with consequent clinical improvement (21).

### Justification

Existing scientific production shows a high rate of disability and morbimortality in people suffering from some psychiatric disorder concerning the rest of the general clinical population, especially in those patients with a severe and long-term mental disorder (LTMD) (1, 12, 14, 24–28), highlighting dysfunctions of the psychotic and affective spectrum: schizophrenia and bipolar disorder, (respectively) (24, 27). This morbidity and mortality rate in the psychiatric population is up to 20% higher and, quantitatively, represents an average of 25 years of life lost (24–27, 29). Besides, patients with LTMD have a life expectancy of less than 20% (57 years in men and 65 years in women) (14, 25). It is estimated that the relative risk of this disease is 2.41 higher for mortality from any causes (24), these being mainly comprised of cardiovascular, infectious, respiratory, and endocrine diseases (60% of premature deaths in this clinical population) (14, 25, 30). Also, the leading established causes of mortality are closely linked to the development of the Metabolic Syndrome (MS) (1, 3, 25–28, 31–33), also called insulin resistance syndrome (24, 33). The MS is considered a determining factor in the physical health of the patient, tripling the incidence of cardio-metabolic diseases (27–29).

The main etiopathogenic determinants of this fact are the factors inherent to the disease itself, as well as genetic factors (3, 24–26, 34) and resistance to adequate care in terms of physical health (27, 33, 35). However, the main modifiable risk factor in the LTMD population lies in the acquisition of unhealthy lifestyles, characterized by high-energy dietary patterns, with high consumption of ultra-processed foods and low fruit and vegetable intake (36). In addition, the psychiatric population has low levels of physical activity, with increased rates of smoking and associated substance abuse (3, 27, 37, 38).

Despite the magnitude and severity of the problem, interventions aimed to modify lifestyles do not play an essential role in therapy and are not part of the usual clinical practice with the psychiatric population (1, 27, 31, 33). This fact could be explained by the lack of understanding of the multiple mechanisms and etiological factors involved in the neurogenesis of schizophrenia (2), and leads to a multidisciplinary approach, but essentially psychopharmacological and psychotherapeutic (33, 39). It is, therefore, vital to address modifiable factors such as dietary patterns, which have evidenced to be an efficient therapeutic intervention to improve both the psychopathological dysfunction and the physical health of the subjects and can be considered as an addition to the conventional therapeutic approach (1, 3, 7, 17, 40).

In this sense, some dietary interventions carried out to modulate intestinal microbiota in psychotic disorders through the use of so-called “psychobiotics” (1, 17–22). This term refers to the set of substances that include probiotics and/or prebiotics and whose administration causes health benefits in psychiatric patients (20–22). Probiotics include microorganisms of the intestinal biota, which, provided in adequate quantities, offer a benefit for the host (highlighting the genera Lactobacillus and Bifidobacterium, among others) (1, 7, 8, 12, 16–21). On the other hand, prebiotics are non-digestible dietary fiber (mainly fructooligosaccharides and oligosaccharides, inulin or pectins) (1, 17), which are substances that promote optimal growth and development of probiotics in the gastrointestinal tract, reducing pathogenic microbiota (7, 12, 15, 19), through the production of short-chain fatty acids (17, 21).

It is worth noting the growing effort to highlight the role played by prebiotics and or probiotics in the microbiota-intestine-brain axis, which is currently a relevant object of study (1, 22, 41).

In this regard, according to Patra (19) and Teasdale et al. (37), adequate dietary planning in psychiatric patients with psychopathological dysfunction and at risk of iatrogenic metabolic syndrome, could be considered as a therapy of choice in these subjects, improving altered clinical patterns and difficulties in the patient’s vital and functional performance. Similarly, adequate nutritional management could be used as an adjunct to antipsychotic pharmacotherapy and the cardio-metabolic approach, reducing the number of homeostatic drugs or even replacing them in cases of intolerance in the target population (14, 39).

In short, the future of the development of Mental Health is determined by the need for a multimodal approach, where nutritional factors represent the cornerstone in achieving optimal results in health, level of functionality, and, therefore, quality of life of patients (35, 42).

Likewise, dietary advice on modulation with high prebiotic and probiotic content has the added value of improving the morbidity and mortality associated with schizophrenia, with optimal levels in terms of cost-effectiveness, better than those shown by the approaches currently used.

## Methods/design

### Study aims

#### Main objective

Determination of the nutritional and cardio-metabolic efficacy of a prebiotic and probiotic dietary intervention in patients with schizophrenia spectrum disorders.

#### Specific objectives

- To determine the baseline nutritional status of the target population.
- To identify the usual dietary patterns in this population, clarifying the nutritional value of the main dishes consumed, as well as their link with the physical health status of individuals.
- To know the existing scientific evidence regarding the construction of determinants (explicit and implicit) that influence the microbiota-intestine-brain axis.
- To evaluate the psychopathological impact of the incorporation of prebiotics and probiotics in the habitual dietetic-nutritional pattern in patients diagnosed with the spectrum of schizophrenia.
- To evaluate the cardio-metabolic impact of a standardized dietary planning with high prebiotic and probiotic content, adapted to the inherent characteristics of the psychiatric population.
- To develop and validate a program that allows for the detection of areas of improvement, establishing assessment strategies, and an appropriate action plan in Mental Health, which allows for adequate dietary care through the use of psychobiotics.

### Study design

A two-arms, double-blind, randomized in balanced blocks clinical trial of 6 months of intervention, will be carried out in psychiatric patients diagnosed with schizophrenic spectrum disorders (without distinction by type). The control group (CG) will be made up of those participants who will receive conventional dietary advice (43) on an individual basis. In the intervention group (IG), this advice will be established individually through intensive nutritional guidance (44) offering a food pattern with a high prebiotic and probiotic content. In both intervention groups, educational material of visual support will be used during the sessions. The dietary intervention will be designed and supervised by qualified personnel with recognized competencies for this intervention (nurses and dietitians), carried out by specialized mental health nurses, and will be agreed upon through serial interviews and focus groups. In this sense, these focus groups will be applied to improve the established dietetic-nutritional intervention, guaranteeing its correct adaptation, according to the study population.

The study will begin with a group session for the presentation of the research project in the health center and/or psychiatric service consultation. During the development of the study, data will be collected on the psychopathological state [Positive and Negative Syndrome Scale (PANSS) (45) and Personal and Social Functioning Scale (PSP) (46) scales; Supplementary Tables 1, 2], and blood test (hemogram, lipid profile, etc.). Measures will be taken at the beginning (basal), at 3 and 6 months. The estimation of intestinal microbiota and the usual nutritional pattern will also be assessed at the beginning and 6 months, using a stool test and a validated Food Frequency Questionnaire (FFQ) (47), respectively. The use of the FFQ will allow us to know both the average intake of grams of fiber, fat, etc. and the frequency of consumption by food groups (with particular attention to fermented foods). However, the main reason for using this tool is that it will allow us to assess changes in dietary patterns over the medium term (39). To evaluate the degree of adherence, participants in the IG will fill a specific weekly record of the main dishes/food consumed with a high prebiotic and/or probiotic content will be measured by the weekly completion (during the 6 months of intervention) of a record that includes the main foods consumed with a high symbiotic value (fermented foods, whole grains, green leafy vegetables, fruit, etc.) (Supplementary Figure 1). This record will be completed by the patients themselves or, in cases of incapacity or lack of autonomy in preparing the dishes consumed by family members or the primary caregiver. At least, anthropometric parameters will also be analyzed monthly (BMI, blood pressure, heart rate, abdominal perimeter) (Supplementary Table 3).

### Selection of participants

**FIGURE 1:**
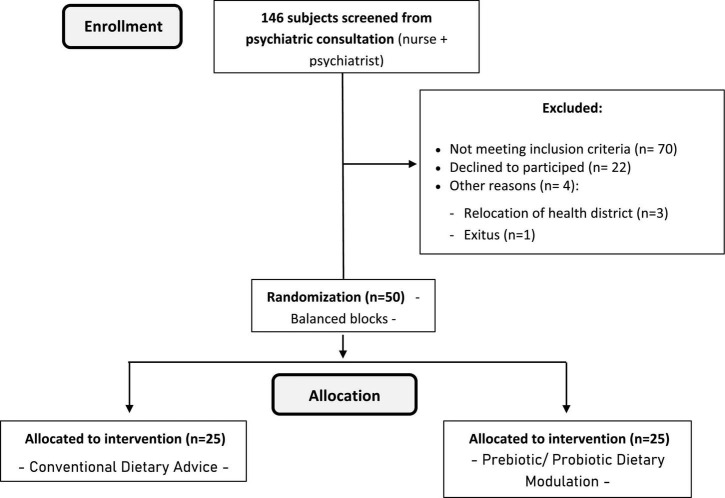
Flow chart of the participants (CONSORT diagram).

For the assessment of the method’s effectiveness, a sample size of 22 individuals has been estimated (11 for the IG and 11 for the CG, with a power of 80% and a confidence of 95%, expecting a risk/prevalence difference of 63% post-intervention (48). The final size of 50 individuals has been established (25 for the IG and 25 for the GC) to minimize the effect of possible losses and the low study completion rates, especially in participants with significant negative symptoms. Participants who express a clear wish to participate voluntarily in the study will be assigned, through randomization in balanced blocks, to the IG or CG (Figure 1). Randomization will be conducted according to the results found in stool culture analysis (balancing the prevalence of dysbiosis in both groups). Concerning the established inclusion/exclusion criteria, these will be:

#### Inclusion criteria

- Patients diagnosed on the spectrum of schizophrenia (without distinction by type), according to criteria DSM-5 and/or ICD-11.
- Age between 18 and 65 years.
- Absence of gastrointestinal comorbidity that contraindicates the use of prebiotics and/or probiotics (intolerance, explosive diarrhea, acute abdominal pain, etc.).
- To show clinical stability for 6 months before the start of the study (absence of psychiatric hospitalization, maintenance of the level of functionality, and lack of social and occupational absenteeism).
- To manifest agreement to participate in the study and to sign of informed consent (Supplementary Appendices I–III).

#### Exclusion criteria

- To suffer from somatic or neurocognitive situation that prevents participation and collaboration in the fulfillment of the protocol.
- To follow standardized dietary planning not modulated by the population under study (catering, institutional or collective feeding, etc.).
- Concomitant administration of antibiotherapy during the intervention phase.
- Refusal to participate in the study.

### Study variables

#### Result variables

##### Clinical efficacy of prebiotic/probiotic dietary modulation

- Scale for Positive and Negative Schizophrenia Syndrome (45) (categorized PANSS): discrete.
- Personal and Social Functioning Scale (46) (categorized PSP) outcome: discrete.

##### Tolerability and modulation of the nutritional dietary pattern

- Food Frequency Questionnaire (FFQ) (47) result: continuous
- Culinary knowledge and responsibility for feeding: nominal.

##### Anthropometric variables and physical health

Weight (kg, continuous), height (cm, continuous), BMI (kg/m^2^, continuous) abdominal circumference (cm, continuous), systolic blood pressure (mmHg, continuous), diastolic blood pressure (mmHg, continuous), heart rate (ppm, discrete).

##### Blood test variables

- *Hematological profile*: Red blood cells (x10e^6^/mm^3^, continuous), hemoglobin (g/dL, continuous), hematocrit (%, continuous), M.C.V. (fL, discrete), H.C.M. (pg, discrete), C.H.C.M. (g/dL, discrete), R.D.W (%, continuous), leukocytes (×10e^3^/mm^3^, discrete), neutrophils (×10e^3^/m, continuous), lymphocytes (×10e^3^/m, continuous), monocytes (×10e^3^/m, continuous), eosinophils (×10e^3^/m, continuous), basophils (×10e^3^/m, continuous), platelets (×0e^3^/mm^3^, discrete), M.P.V. (fL, discrete). See Table 1 for abbreviations corresponding to the blood test variables.

**TABLE 1:** Blood test variables: Abbreviations.

	Abbreviation	Meaning
Hematological profile	M.C.V	Mean corpuscular volume
	H.C.M	Mean corpuscular hemoglobin
	M.C.H.C	Mean corpuscular hemoglobin concentration
	R.D.W	Red cell distribution width
	M.P.V	Mean platelet volume
Biochemical profile	ALT	Alanine transaminase
	GPT	Glutamic pyruvic transaminase enzyme
	G-GT	Gamma-glutamyl transferase
	ALP	Alkaline phosphatase
	Na^+^	Sodium
	K^+^	Potassium
	Cl^–^	Chlorine
	Ca^2+^	Calcium
	HbA1c	Glycosylated hemoglobin
	IFCC	International federation of clinical chemistry and laboratory medicine
	Fe^2+^	Iron
	FRT	Ferritin
	LDH	Lactate dehydrogenase
	C-HDL	High-density lipoprotein
	C-LDL	Low-density lipoprotein
	TSH	Thyroid stimulating hormone
	PRL	Prolactin
	LUES	Syphilis
	HCV	Hepatitis C virus
	HAV	Hepatitis A virus
	HIV	Human immunodeficiency virus

- *Biochemical profile.* ALT/GPT (IU/L, discrete), G-GT (IU/L, discrete), FAL (IU/L, discrete), Na^+^/K^+^ (mEq/L, discrete/continuous, respectively), Cl^–^ (mEq/L, continuous), Ca^2+^ (mEq/L, continuous), urate (mg/dL, continuous), glucose (mg/dL, discrete), HbA1c (%, continuous), HbA1c IFCC (mmol/mol, continuous), fructosamine (mcmol/L, discrete), creatinine (mg/dL, continuous), Fe^2+^ (mcg/dL, discrete), FRT (mcg/dL, discrete), folate (mcg/dL, continuous), vit.B12 (ng/mL, discrete), vit.D total (D2 + D3) 25-OH (ng/mL, discrete) cholesterol (mg/dL, discrete), triglycerides (mg/dL, discrete), LDH (IU/L, discrete), C-HDL (mg/dL, discrete), C-LDL (mg/dL, discrete), total cholesterol/C-HDL (mg/dL, discrete), glomerular filtrate estimation (mL/m/173, discrete), TSH (mU/L, continuous), PRL (ng/dL, continuous), LUES (IU/L, nominal/discrete), a-HAV-M (IU/L, nominal/discrete), a-HCV (IU/L, nominal/discrete), HBsAg (UI/L, nominal/discrete), a-HBC-IgG (UI/L, nominal/discrete), a-HBs (UI/L, continuous), a-HIV (mcL, nominal/discrete).

#### Stool variables

##### Stool culture

General bacteriology. Usual mixed flora: Lactobacillus (CFU/g, continuous), Bifidobacterium (CFU/g, continuous)//Disbiosis: *Salmonella* spp. (nominal, Presence/absence), *Shigella* spp. (nominal, presence/absence), *Yersinia* spp. (nominal, presence/absence), Hafnia alvei (nominal, presence/absence), *Aermonas* spp. (nominal, presence/absence), *Campylobacter* spp. (nominal, presence/absence).

### Independent variables

#### Sociodemographic variables

Age (continuous), gender (nominal), legal representative (nominal), household composition (nominal), economic level (ordinal), level of studies (ordinal), area of residence (nominal).

#### Therapeutic variables

Previous antipsychotic (nominal), the dose of antipsychotic (mg, continuous), the reason for a change in antipsychotic treatment (nominal).

#### Clinical variables

Type of psychotic disorder (nominal), duration of illness (continuous), age of first hospitalization (continuous), number of previous hospitalizations (discrete), number of previous relapses (discrete), number of previous suicidal behaviors (discrete), number of subsequent hospitalizations (discrete), number of subsequent relapses (discrete), number of subsequent suicidal behaviors (discrete), number of subsequent unscheduled consultations (discrete), substance abuse (nominal), type of substance (nominal), associated cardio-metabolic diagnosis (nominal).

Anthropometric measurements will be collected following the recommendations of the Manual of Standardized Anthropometry (49). Weight, height, and BMI shall be measured with an SECA^®^ 703s stadiometer and scale, with an accuracy of 0.1 kg and 0.1 cm, respectively. The abdominal perimeter shall be determined at the midpoint between the last rib and the iliac crest at the end of a normal expiration. The WelchAllyn^®^ ProBP 2,400 digital sphingomanometer shall be used for the study of blood pressure and heart rate.

### Statistical analysis

Quantitative variables will be presented with mean and standard deviation, and qualitative variables will be shown in frequencies and percentages.

The Kolmogorov-Smirnov test will be used to compare the goodness of fit to a normal distribution of data from quantitative variables. For the contrast of bivariate hypotheses, the Student *t*-test will be performed for two means, while for qualitative variables, the Chi-Square and Fisher’s exact test will be used, when necessary. Likewise, for the analysis of three or more means, the ANOVA of repeated means will be used. On the other hand, the correlation between quantitative variables will be verified through the Pearson’s coefficient r. When the normality or homoscedasticity criteria are not met, non-parametric versions of the above tests will be performed.

Logistic regressions will be carried out to determine which variables can determine the improvement of the nutritional pattern and physical health through the FFQ (47), as well as blood and stool analytical values. Similarly, this analysis will be established concerning the psychopathological status through the PSP (46) and PANSS (45) scales, both of which have a discrete quantitative and nominal result values, according to established cut-off points and clinical interpretation. Raw and adjusted odds ratios will be calculated. Log-likelihood, the goodness of fit statistic, Cox and Snell R2, Nagelkerke R2, and Hosmer-Lemeshow tests should be used to assess the overall model fit. The exponentiation will be used to calculate the beta coefficients.

These multivariate tests will allow us to identify and adjust for the possible confounding effect of the independent variables on the outcome variables.

For all statistical analyses, an alpha error probability of less than 5% (*p* < 0.05) will be accepted, and the confidence interval will be calculated at 95%. The software SPSS (version 25.0) and EPIDAT (version 4.2) shall be used for the statistical analysis.

### Work plan

An intervention schedule has been established, with a total duration of 6 months, which is divided into three blocks:

#### Block 1

This first block focuses on the selection of the target population according to inclusion criteria. Firstly, a group session to present the program and the methodology of the study will be carried out. During the first 15 days of the study, a focus group with professionals to reach a consensus on the intervention will be held. Subsequently, the appropriate modifications will be made to improve and adapt to the dietary and nutritional intervention.

Consequently, the recruitment and initial psychopathological and nutritional assessment of the participants will be carried out, using the PANSS (45) and PSP (46) scales. For the nutritional evaluation, the analytical and anthropometric basal determination of the participating patients will be carried out, as well as the evaluation of the habitual dietary pattern through a validated FFQ (47) and weekly record of the main dishes and foods consumed. Similarly, an estimate of the intestinal bacterial flora is required employing stool culture.

#### Block 2

The second block includes the implementation of the 6-month individual nutrition education program (associated with 2 months of educational reinforcement, according to block 3). It will consist of eight sessions, the first four being biweekly, followed by 4 monthly, to which four sessions of educational reinforcement will be added to the 3 and 5 months of study. The minimum duration of each session has been established for 30 min. However, this length could be different, considering the characteristics of the participants. The control group will be made up of those participants who will receive standardized dietary advice (35). In this sense, the education content in the intervention group will be based on general principles of conventional dietary advice in an intensified manner (36), centered on the acquisition of specific knowledge about: (I) Underlying mental pathology, lifestyles and associated comorbidities; (II) Immediate principles: Carbohydrates, lipids, proteins, fiber, vitamins, and minerals; energy needs; consumption requirements; (III) Water requirements; (IV) Foodstuff; (V) Description and justification of prescribed prebiotic and probiotic diet; (VI) Culinary techniques: conservation of properties of the prebiotic and probiotic diet; (VII) Optimal distribution and interchange of foods with high prebiotic and probiotic content; (VIII) Feeding in particular situations.

In both the IG and the CG, visual support resources will be used during the development of the established sessions.

#### Block 3

Finally, to evaluate the effectiveness of the intervention, the modification in the nutritional, the cardio-metabolic, and the psychopathological area will be assessed. For doing this, researchers will carry out anthropometric determination, clinical evaluation, the performance of stool culture, and the study of the dietary pattern, as we commented above.

Likewise, in this block, an educational reinforcement (both in IG and CG) of what was treated in Block 2 will be offered, 3 and 5 months after the beginning of the block, every 15 days for the IG and monthly for the CG.

Once the intervention is concluded, the analysis of the collected data will be performed, culminating in the development of the scientific production and the writing of the research report.

## Discussion

Firstly, it is expected to obtain the necessary information for the determination of the optimal dietary pattern for those participants in the study, thus allowing the development of a nutritional intervention with high prebiotic and probiotic content, appropriate for the population under study.

Likewise, the aim is to ensure that all participants improve their health status through the adaptation of the feeding pattern, developing adherence to healthier lifestyles adapted to the conditions of each patient.

Finally, it is expected to demonstrate that an adequate dietary modulation with a high prebiotic and probiotic content, leads to a significant improvement in the nutritional status and, therefore, the cardio-metabolic, of the participants, mediated by the microbiota-intestine-brain axis. Furthermore, it is presumed to reach a degree of evidence that allows establishing nutritional management as an effective therapeutic intervention in the psychopathological treatment of patients with schizophrenia spectrum disorders, in any of its variants.

### Limitations

Potential limitations lie in the sample size of subjects included during the recruitment phase, the possible loss or non-cooperation of participants, and the loss or non-cooperation of participants in the intervention phase (especially those subjects with a predominance of negative schizophrenia symptomatology). This fact can lead to a possible lack of representativeness of the target population. Thus, to reverse this situation, the recruitment of the target population will be increased (more than doubled).

Likewise, the lack of capacity to prescribe symbiotic formulas and placebo management by mental health nurses, according to the legislative context in which the study will be carried out, prevents us from knowing the accurate range that an intervention with a high symbiotic content could have on these patients. Nevertheless, it is essential to emphasize that the main objective of the intervention is to assess the efficacy of a strategy based on health education. This tool is very cost-effective and is available for nursing professionals. Furthermore, from our perspective, this intervention can significantly impact the lifestyle habits of these patients in a sphere that has been forgotten as part of the traditional treatments.

Finally, the available evidence on the object of study makes it difficult to contrast the results obtained in different contexts of application.

## Ethics statement

The study will be carried out respecting the fundamental principles established in the Declaration of Helsinki (1964)50, the Council of Europe Convention on Human Rights and Biomedicine (1997)51, the UNESCO Universal Declaration on the Human Genome and Human Rights (1997)52. The research will also follow the requirements established by Spanish legislation (Organic Law 3/2018 of 5 December and Law 41/2002 of 14 November). This study protocol has been registered on the platform clinicaltrials.gov (NCT04366401). This research also has the permission of the Zamora Health Area Drug Research Ethics Committee at the Regional Government of Castile and León, Spain (No. reg. 468). All the information analyzed by the principal investigator of this study is subject to the maintenance of professional secrecy. In any case, each participant will be assigned a code as a registry, where all the comparative data will be mechanised in an anonymous way, delimiting the access to the database only to the personnel linked to the development of the study, previous authorisation of the investigator in charge of it.

## Author contributions

AS-J, GM-R, MG-R, and MR-S contributed to the conception and design of the study. AS-J, GM-R, JAG-M, RM-L, and MR-S contributed to the acquisition, analysis, and interpretation of results. AS-J and GM-R drafted the manuscript. AS-J, GM-R, and MR-S critically revised the manuscript. All authors contributed and approved the submitted version and agreed to be accountable for all aspects of the work, ensuring integrity and accuracy.
