# Supplementary material for: Efficacy of nutrition education for the increase of symbiotic intake on nutritional and metabolic status in schizophrenic spectrum disorders: A two-arm protocol
Source: Front Nutr. 2022 Aug 10;9:912783. doi: 10.3389/fnut.2022.912783 (PMC9399917; doi:10.3389/fnut.2022.912783)
Supplement: Supplementary file 1 [file Data_Sheet_1.pdf]

# - SUPPLEMENTARY MATERIAL -

The Model Consent Form and other related documentation provided to participants and authorized substitutes are presented.

The dietary intervention was designed and supervised by qualified personnel with recognized competencies for this type of intervention (nurses and dietitians) and will be agreed upon through serial interviews and focus groups. In this sense, these focus groups will be applied to improve the established dietetic-nutritional intervention, guaranteeing its correct adaptation, according to the study population.

During the development of the study, data of multiple variables are collected (sociodemographic, clinical, therapeutic..) as well as the psychopathological state: Positive and Negative Syndrome Scale -PANSS and Personal and Social Functioning Scale -PSP-, scales. The measures scales will be taken at the beginning (basal), at three and six months.

- Positive and Negative Syndrome Scale -PANSS-: It provides four dimensional scores: Positive syndrome, Negative syndrome, Compound scale, General psychopathology.

  The score on the positive (PANSS-P), negative (PANNS-N), and general psychopathology (PANSS-PG) scales is obtained by adding up the scores of each item. The scores will therefore range from 7 to 49 for the positive and negative scales, and from 16 to 112 for the general psychopathology.

  The score on the composite scale (PANNS-C) is obtained by subtracting the score on the negative scale from the score on the positive scale. This scale can have a positive or negative valence, ranging from -42 to + 42.

  There are no cut-off points for the direct scores obtained, but these are transformed by means of a conversion table into percentiles.

- Personal and Social Functioning Scale -PSP-: Scoring interval (range 10 points: 0-100) in relation to the degree of dysfunction of the areas: self-care, personal and social relationships, socially functional activities (work/study), disturbing and aggressive behaviour. To establish the final decimal point of this interval, 10 functional aspects must be scored (YES: 1 point, NO: 0 points). Finally, the higher the score, the better the patient's functional level.

To evaluate the degree of adherence, the participants in the GI will fill in a specific weekly register of the main dishes/foods consumed (symbiotic register) and will receive nutritional information during 6 months of intervention. At least, anthropometric parameters will also be analyzed monthly (BMI, blood pressure, heart rate, abdominal perimeter), and blood test (hemogram, lipid profile, etc.). Measures will be taken at the beginning (basal), at three and six months.

The estimation of intestinal microbiota pattern will also be assessed at the beginning and six months.

Supplementary Table 1 - **POSITIVE AND NEGATIVE SYNDROME SCALE *(PANSS)* -**

Escala Positiva (PANSS-P)							
Delusions	1	2	3	4	5	6	7
Behavioral Disorganization	1	2	3	4	5	6	7
Hallucinations	1	2	3	4	5	6	7
Excitement	1	2	3	4	5	6	7
Grandiosity	1	2	3	4	5	6	7
Suspicion/Judgment	1	2	3	4	5	6	7
Hostility	1	2	3	4	5	6	7

Total PANSS-P	

Escala Negativa (PANSS-N)							
Blunted Affect	1	2	3	4	5	6	7
Emotional Withdrawal	1	2	3	4	5	6	7
Poor Contact	1	2	3	4	5	6	7
Social Withdrawal	1	2	3	4	5	6	7
Abstract Thinking	1	2	3	4	5	6	7
Lack of Spontanely / Conversation Flow	1	2	3	4	5	6	7
Stereotypical Thinking	1	2	3	4	5	6	7

Total PANSS-N	

Composite Index (Total PANSS-P)-(Total PANSS-N)	

Psicopatología General (PANSS-PG)							
Somatic Concern	1	2	3	4	5	6	7
Anxiety	1	2	3	4	5	6	7
Guilt Feelings	1	2	3	4	5	6	7
Tension	1	2	3	4	5	6	7
Mannerisms/Posturing	1	2	3	4	5	6	7
Depression	1	2	3	4	5	6	7
Motor Retardation	1	2	3	4	5	6	7
Uncooperativeness	1	2	3	4	5	6	7
Unusual Thoughts Content	1	2	3	4	5	6	7
Disorientation	1	2	3	4	5	6	7
Poor Attention	1	2	3	4	5	6	7
Lack of Insight	1	2	3	4	5	6	7
Disturbance of Volition	1	2	3	4	5	6	7
Poor Impulse Control	1	2	3	4	5	6	7
Self-development	1	2	3	4	5	6	7
Socially Active Avoidance	1	2	3	4	5	6	7

Total PANSS-PG	

- POSITIVE SYNDROME		POINTS		PERCENTIL
- NEGATIVE SYNDROME		POINTS		PERCENTIL
- COMPOSITE INDEX		POINTS		PERCENTIL
- GENERAL PSYCHOPATHOLOGY		POINTS		PERCENTIL

- RESTRICTIVE SYSTEM	
- INCLUSIVE SYSTEM	

Attention: This document contains confidential patient information, so it should be kept by the principal investigator along with the rest of the study documentation.

- OBSERVATIONS:

Attention: This document contains confidential patient information, so it should be kept by the principal investigator along with the rest of the study documentation.

- MANAGEMENT STANDARDS AND SCORING –

✓ Administration:

- It must be administered by a clinician, using the semi-structured interview technique.
- The approximate time of administration is 30-40 minutes.

✓ Score:

- It provides four dimensional scores:

  1. Positive syndrome.
  2. Syndrome negative.
  3. Compound scale.
  4. General psychopathology.

- The score on the positive (PANSS-P), negative (PANNS-N), and general psychopathology (PANSS-PG) scales is obtained by adding up the scores of each item. The scores will therefore range from 7 to 49 for the positive and negative scales, and from 16 to 112 for the general psychopathology.

- The score on the composite scale (PANNS-C) is obtained by subtracting the score on the negative scale from the score on the positive scale. This scale can have a positive or negative valence, ranging from -42 to + 42.

- There are no cut-off points for the direct scores obtained, but these are transformed by means of a conversion table into percentiles.

- The following table represents the direct scores corresponding to the 5th, 25th, 50th, 75th and 95th percentiles.

PERCENTIL	PANSS-P SCORE	PANSS-N SCORE	PANSS-C SCORE	PANSS-PG SCORE
5	12	9	-24	28
25	21	19	-10	38
50	26	25	1	45
75	31	35	10	52
95	38	43	19	61

- In addition to the dimensional score discussed in the previous points, the PANSS also provides categorical information, indicating whether the schizophrenic disorder is positive, negative or mixed. For this purpose, there are two more or less restrictive systems.

  I. The most restrictive: to establish the type of schizophrenic disorder, use the scores obtained in the positive and negative scale items. He considers that schizophrenic disorder is:

     1. *Positive:* when on the positive scale more than 2 items score more than 3 and on the negative scale less than 3 items score more than 3.

2. ***Negative:*** when in the negative scale more than 2 items get a score above 3 and in the positive scale less than 3 items get a score above 3.

3. ***Mixed:*** when in both scales there are more than 2 items that get scores above 3.

II. The least restrictive or inclusive: to establish the type, it uses the score obtained on the composite scale, and more specifically its valence, so that it considers the schizophrenic disorder to be:

1. Positive when the valence on the composite scale is +.
2. Negative when the valence on the composite scale is -.

Attention: This document contains confidential patient information, so it should be kept by the principal investigator along with the rest of the study documentation.

Supplementary Table 2 - PERSONAL AND SOCIAL PERFORMANCE (PSP) -

1.- Rate the degree of dysfunction of the patient during the last month in the following 4 main areas (mark an -X- in the usual situation). To determine the level of dysfunction you must use the operational criteria provided below. Note that there are common criteria for areas a-c and other specific criteria for area d.

		Absent	*Mild*	*Obvious*	*Marked*	*Severe*	*Very Serious*
a.	*Self-care*	☐	☐	☐	☐	☐	☐
b.	*Personal and social relations*	☐	☐	☐	☐	☐	☐
c.	*Activities in functional areas: work/study*	☐	☐	☐	☐	☐	☐
d.	*Disruptive and aggressive behaviors*	☐	☐	☐	☐	☐	☐

* Severity levels in areas a-c

(I) ***Absent.***
(II) ***Mild:*** Only known to someone very close to the person.
(III) ***Obvious:*** Difficulties clearly identifiable by others, but which do not substantially interfere with personal skills to exercise their role in each area, taking into account the socio-cultural context, age, sex and educational level of the person
(IV) ***Marked:*** difficulties that strongly interfere with the performance of roles in each area; however, the person is still able to do some things without professional or social help, although inadequately and/or occasionally; if helped, he or she is able to reach his or her previous level of functioning.
(V) ***Severe:*** Difficulties that render the person unable to perform any function in each area if not professionally assisted, or present a destructive pattern, but without survival risks.
(VI) ***Very serious:*** impairments and difficulties so severe that they put the person at risk of survival

** Gravity levels in area d

(I) ***Absent.***
(II) ***Mild:*** Slight rudeness, unsociability or complaints.
(III) ***Obvious:*** Speaking too loudly or in too familiar a manner, or eating in a socially unacceptable manner
(IV) ***Marked:*** Insulting others in public, or breaking objects, often acting in a socially inappropriate but not dangerous manner (e.g., undressing or urinating in public).
(V) ***Severe:*** Threats of frequent verbal or physical assault, without intent or possibility of serious injury.
(VI) ***Very Serious:*** Frequent acts of aggression, directed or likely to cause serious injury.

2.- Select a 10-point interval. The selection of this interval is based on degrees of dysfunction that have been determined for the 4 main areas: a) self-care; b) personal and social relationships; c) socially functional activities: work/study; d) disruptive and aggressive behaviors.

100-91 Excellent performance in all areas. Is recognized for his or her good qualities, deals adequately with life's problems, is involved in a wide range of activities and interests.
90-81 It works well in all areas, it just presents common problems and difficulties.
80-71 Slight difficulties in one or more areas **a-c**.
70-61 Obvious, but not marked difficulties in one or more areas **a-c**, or mild difficulties in - **d** -.
60-51 Marked difficulties in one of the areas **a-c**, or evident difficulties in - **d** -.
50-41 Marked difficulties in 2 or more areas **a-c**, or severe difficulties in one of the areas **a-c**, with or without evident difficulties in **- d -.**
40-31 Serious difficulties in one area and marked in at least one other area a-c, or difficulties marked in **- d -.**
30-21 Severe difficulties in 2 of the areas **a-c**, or severe difficulties in **-d-** with or without deterioration in areas **a-c.**

Attention: This document contains confidential patient information, so it should be kept by the principal investigator along with the rest of the study documentation.

20-11 Severe difficulties in all areas **a-d**, or very severe in - **d** - with or without deterioration in areas **a-c**. If the person reacts to external cues, a score of 16-20 is suggested; if not, a score of 11-15 is suggested.

10-1 Lack of autonomy in basic functioning with extreme behaviours but without risk of survival (6-10) or with risk of survival (1-5), for example: risk of death due to malnutrition, dehydration, infections, or inability to recognize situations of evident danger.

3.- Adjustment within the 10-point range.

The level of dysfunction in other areas should be taken into account when adjusting the score within the decimal range (e.g., 31 to 40), such as:

- Physical and psychological health care
- Accommodation, living area, housing care
- Contribution to household activities, participation in family life or day centre/residential life
- Intimate and sexual relationships
- Child care
- Social network, friends and collaborators
- Adjustment of social standards
- General interests
- Use of transport, telephone
- Coping strategies in crisis situations

4.- Record, between 0-100, the **final score**: [ ]

- OBSERVATIONS:

____

____

____

____

Attention: This document contains confidential patient information, so it should be kept by the principal investigator along with the rest of the study documentation.

Supplementary Table 3 - ANTHROPOMETRIC ASSESSMENT AND PHYSICAL HEALTH RECORD -

[BASAL]		- MONTH No. 1-		- MONTH No. 2-		- MONTH No. 3-	
**ANTHROPOMETRIC ASSESSMENT**							
- **WEIGHT** (kg):	- **ABDOMINAL GIRTH** *(cm):*	- **WEIGHT** (kg):	- **ABDOMINAL GIRTH** *(cm):*	- **WEIGHT** (kg):	- **ABDOMINAL GIRTH** *(cm):*	- **WEIGHT** (kg):	- **ABDOMINAL GIRTH** *(cm):*
- **SIZE** *(cm):*	- **BMI** *(weight/height^2)*	- **SIZE** *(cm):*	- **BMI** *(weight/height^2)*	- **SIZE** *(cm):*	- **BMI** *(weight/height^2)*	- **SIZE** *(cm):*	- **BMI** *(weight/height^2)*
**CARDIOVASCULAR CONTROL**							
- **BLOOD PRESSURE** *(mmHg):*	- **HEART RATE** *(ppm):*	- **BLOOD PRESSURE** *(mmHg):*	- **HEART RATE** *(ppm):*	- **BLOOD PRESSURE** *(mmHg):*	- **HEART RATE** *(ppm):*	- **BLOOD PRESSURE** *(mmHg):*	- **HEART RATE** *(ppm):*

- **OBSERVATIONS:**

Attention: This document contains confidential patient information, so it should be kept by the principal investigator along with the rest of the study documentation.

- MONTH No. 4 -		- MONTH No. 5 -		- MONTH No. 6 -	
**ANTHROPOMETRIC ASSESSMENT**					
- **WEIGHT** (kg):	- **ABDOMINAL GIRTH** *(cm):*	- **WEIGHT** (kg):	- **ABDOMINAL GIRTH** *(cm):*	- **WEIGHT** (kg):	- **ABDOMINAL GIRTH** *(cm):*
- **SIZE** *(cm):*	- **BMI** *(weight/height²)*	- **SIZE** *(cm):*	- **BMI** *(weight/height²)*	- **SIZE** *(cm):*	- **BMI** *(weight/height²)*
**CARDIOVASCULAR CONTROL**					
- **BLOOD PRESSURE** *(mmHg):*	- **HEART RATE** *(ppm):*	- **BLOOD PRESSURE** *(mmHg):*	- **HEART RATE** *(ppm):*	- **BLOOD PRESSURE** *(mmHg):*	- **HEART RATE** *(ppm):*

- **OBSERVATIONS:**

Attention: This document contains confidential patient information, so it should be kept by the principal investigator along with the rest of the study documentation.

I'VE EATEN THIS WEEK

WEEK ________

Mark with an x the options you have included in your daily menu.

	M	T	W	T	F	S	S	TOTAL DAYS
1 salad plate with vegetables of different colours.								
1 plate of cooked vegetables (steamed, boiled, sautéed, baked, etc.).								
At least one piece of fruit.								
More than one piece of fruit.								
Cereals such as oats or rye, either as whole grain bread, in homemade pastries or mixed with milk or yogurt.								
At least one natural yogurt.								
More than one natural yogurt.								
Other types of fermented dairy such as cottage cheese, fresh cheese, milks fermented or kefir.								

Attention: This document contains confidential patient information, so it should be kept by the principal investigator along with the rest of the study documentation.

**A plate of vegetables (lentils, beans, chickpeas, broad beans or peas).**								
**A stew prepared with onion and tomato sauce (it can be vegetables).**								
**A handful of raw nuts.**								

THE STAR FOODS

These foods contain the largest amount of fiber fermentable by the bacteria in our colon. Try to include at least 1 each day.

VEGETABLES:

- Onion
- Asparagus
- Artichokes
- Leek
- Tomato
- Cucumber
- Carrot

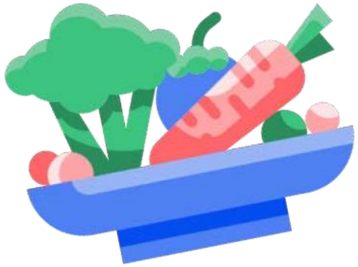

FRUITS:

Banana; Apple (better with skin);
Orange; Tangerine; Blueberries; Grapes

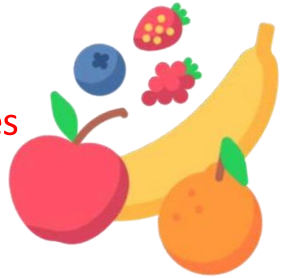

LEGUMES:

- Beans
- Peas

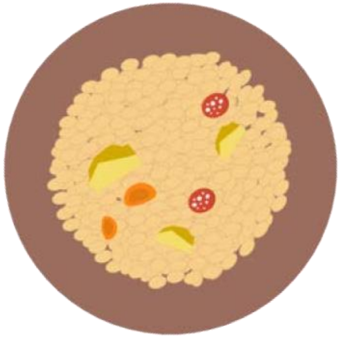

CEREALS:

- Oats
- Rye

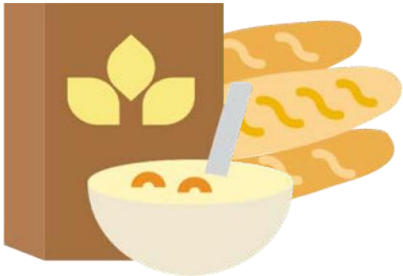

HEALTHY EATING RECOMMENDATIONS

You must eat at least 2 portions of vegetables a day. One of them should be in the form of raw vegetables, for example, in salad. Remember to include vegetables of different colours.

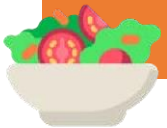

Includes vegetables 2 to 3 times a week. These can be eaten in stews, in stir-fries with vegetables, or in salads. You can also eat them as a garnish for meat, fish and eggs.

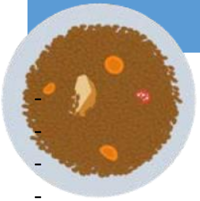

Eat 2 to 3 pieces of fresh fruit every day.

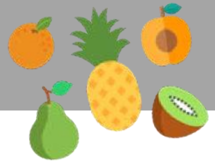

Remember to include 2 to 3 portions of dairy every day, and try to have at least one portion of fermented dairy such as yogurt, fresh cheese or kefir.

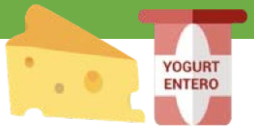

Always choose water as a drink at your meals.

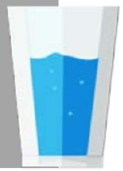

Try to always eat your whole grain. Although they provide us with the same amount of energy, they contain a greater amount of fiber and nutrients that are very necessary for the maintenance of health.

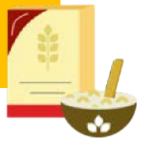

You should consume between 3 and 4 portions of fish per week, alternating between white (hake, cod, monkfish, sole, etc.) and blue (anchovy, sardine, salmon, emperor, tuna).

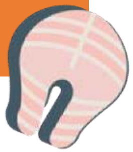

Try to cook and dress your salads always with extra virgin olive oil.

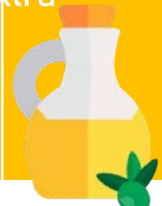

HEALTHY EATING RECOMMENDATIONS

AVOID:

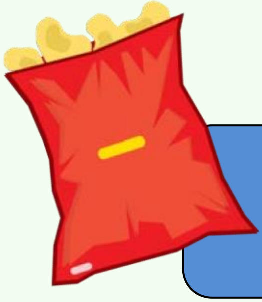

Highly processed foods such as ready meals, sauces, snacks and pastries

The juices, even if they are natural.

They provide too much sugar

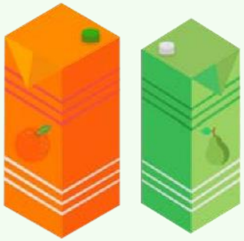

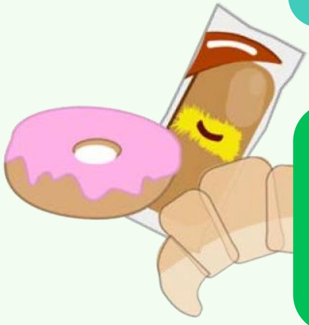

Sugar, pastries and sweet desserts, especially if they are industrial. If you eat them, make sure they are only eaten on special occasions and prepared at home to ensure that quality ingredients have been used.

Sugared soft drink.

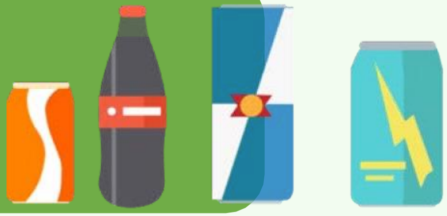

HEALTHY EATING RECOMMENDATIONS

	MONDAY	TUESDAY	WEDNESDAY	THURSDAY	FRIDAY	SATURDAY	SUNDAY
BREAKFAST	Coffee Yogurt with oats and blueberries	Tea<br>Toasted flour<br>wholemeal rye with oil, tomato and ham	Oatmeal coffee with milk, banana, almonds and pure cocoa	Wholemeal Tea with Avocado and slices of tomato	Coffee<br>Wholemeal rye toast with butter and jam without sugar	Tea<br>Yogurt with oats and grapes	Coffee Homemade carrot cake with wholemeal
MID-MORNING	Banana	Apple	Ham rolls cooked with rocket and cottage cheese	Cottage cheese with strawberries	Turkey rolls with guacamole and tomato	Baked apple with cinnamon	Handful of almonds
LUNCH	Salad with tuna and avocado<br>Stewed beans with vegetables<br>Orange	Skipped from leeks and asparagus with prawns<br>Baked chicken leg with baked potato<br>Pear	Chickpea hummus with carrot sticks<br>Grilled salmon with spinach<br>2 Tangerines	Potato omelette with sautéed peppers<br>Apple	Sauteed peas with onions<br>Chicken wings with<br>Banana<br>wine	Salad with cured cheese<br>Stewed potatoes with chicken Infusion	Lettuce hearts with anchovies<br>Paella Orange
SNACK	1 handful of nuts	handful of cashew nuts	Infusion	A handful of toasted almonds	Natural yogurt with cashew nuts	Banana	Coffee with milk
DINNER	Cream of zucchini with grated parmesan cheese<br>Grilled Hake<br>Natural Yogurt with cinnamon	Salad of cherry tomatoes, fresh cheese and black olives<br>French omelette	Sauteed mushrooms with garlic<br>Grilled turkey<br>Natural yogurt	Spinach and goat cheese salad Sole<br>Natural yogurt	Salad with hard-boiled egg and mackerel<br>Orange	Sautéed artichokes with garlic and paprika French omelette<br>Natural yogurt	Sautéed spinach with pine nuts<br>Grilled pork fillet<br>Natural yogurt

- Supplementary Appendix I –

PATIENT / LEGAL REPRESENTATIVE INFORMATION SHEET

Title: EFFICACY OF DIETARY MODULATION WITH SIMBIOTIC CONTENT ON NUTRITIONAL AND METABOLIC STATUS IN SCHIZOPHRENIA SPECTRUM DISORDERS.

Dear Mr/Ms

The study in which you are invited to participate consists of providing reliable and accurate information on the eating habits and pattern of patients diagnosed with Schizophrenia. This will allow the establishment of adequate food and nutrition guidelines rich in prebiotic content, with the ultimate aim of being able to evaluate the impact of this diet on clinical remission and tolerability, as well as cardio-metabolic control in participants.

If you decide to accept to participate in this study, you will need to give your express consent by signing the attached document, a copy of which will be given to you and on which you may request information at any time in the event of any doubt arising or when you consider it appropriate, from the principal investigator or the referring doctor. Please read carefully the attached document provided below.

STUDY OBJECTIVES

The aim of this study is to establish dietary guidelines, rich in prebiotic content, for the improvement of nutritional and cardio-metabolic status, as well as remission and clinical tolerability, in subjects affected by Schizophrenia, in any of its variants.

STUDY DESCRIPTION

- In order to achieve the established objectives, a study has been designed in which all those people diagnosed with Schizophrenia, in any of its variants, can participate.

- The data required for the study will be collected during the scheduled consultations established with the referring doctor or main researcher, and no identifying parameters of the participating person will be included.

- Written consent by signing the attached document is required as an indispensable requirement for participation in this study (Appendix I and II).

- Their participation in this research project requires the performance of additional diagnostic or functional clinical tests (blood analysis, anthropometric determination and serial physical health), as well as the completion of certain assessment scales and

questionnaires related mainly to their diet and, to a lesser extent, to their health and lifestyles. All this information is necessary for the adequate control of the main underlying pathology and physical health, as well as for the evaluation of the individualized dietary pattern of the participant.

- If you accept, you must attend a pre-established number of visits, which may be complementary to those normally carried out in the health care received.

STUDY RISKS

- Since this study involves the collection of analytical and anthropometric data and physical health variables (blood pressure and heart rate), as well as an evaluation of the dietary and nutritional pattern, it is not expected that their participation could involve more risks than those of the diseases they currently suffer from.

- As invasive measures, it is worth highlighting the possible side effects derived from the blood extraction that is required. Such effects are rarely established but, if they do appear, they could include: dizziness, pain or the formation of haematomas, among others.

STUDY BENEFITS

- The results obtained from the study will provide health professionals with useful information on possible aetiological conditions of schizophrenia, as well as establishing therapeutic strategies based on the nutritional pattern and the microbiota-intestine-brain axis. This information could be useful for the benefit of future patients.

ADVANCES IN KNOWLEDGE

- The results obtained from the development of the study will be conveniently disseminated in scientific communications and publications, so that any progress or new discovery made during the course of the study will be made available to you. Your identity will not be disclosed at any time.

VOLUNTARY INVOLVEMENT

- Your participation is voluntary. You are under no obligation to participate if you do not wish to do so.

- You may refuse to participate in the study or may leave it at any time, without prejudice or loss of your rights. If you withdraw from this study, you should only inform your referring physician and/or principal investigator. If you decide not to participate or to leave the study, this will not adversely affect your health care or your participation in future research studies.

- In the event that you withdraw from the study, no new data will be added to the study database, although data that has already been obtained may be used.

CONFIDENTIALITY

- The personal and health data recorded in your medical record and collected for the purposes of this study are those necessary to cover the objectives of the study.

- In the results report, your data will be used anonymously in the form of aggregated data, which will be used exclusively for the purposes of the study. This data cannot be identified as it does not contain any personal information that could reveal your identity.

- The data that may be collected during the study will be processed and analysed with the main purpose of being scientifically evaluated. If you decide to participate in this study, you will be expressly consenting to the processing of your personal and health data. You should be aware that you may exercise your rights of access, rectification, cancellation and opposition at any time by contacting the principal investigator or responsible physician who is treating you.

- It is guaranteed that personal data will be treated with absolute confidentiality. The study will be conducted in accordance with the fundamental principles set out in the Helsinki Declaration (1964), the Council of Europe Convention on Human Rights and Biomedicine (1997), the Declaration Universal Declaration on the Human Genome and Human Rights (1997), as well as the correct fulfilment of the requirements established by Spanish legislation, in accordance with Organic Law 3/2018, of 5 December, on the Protection of Personal Data and the Guarantee of Digital Rights and Law 41/2002, of 14 November, which is the basic law regulating patient autonomy and rights and obligations in the field of clinical information and documentation. All the information analysed by the principal investigator of this study is subject to the maintenance of professional secrecy.

PRELIMINARY INSTRUCTIONS

- Your response is individual. Therefore, we ask that no family member or relative participate in each of the questions without prior consultation or discussion with them.

- It is very important to read and follow in detail the previous instructions of each questionnaire or register provided. Most questions have several answer options, and you should indicate the one you consider best describes your current situation, choosing only one option from the possible answers available. Similarly, there is a need for written description and annotation by numerical value.

- It is very important that all the questions are answered, since many of them are treated together, losing information and validity for the analysis of the results.

CONTACT PERSON

- The principal investigator must answer all questions raised after reading this document. If you require information on new questions during the course of the study, on the need for clinical care arising from the intervention, or on your rights as a participant in this research project, you may contact the responsible investigator or referring physician as necessary.

If you have any questions now or at any time during the course of the study, please contact:

Sr. XXXX XXXXX XXXX

- ***Address:***
- ***Telephone (corporate):***
- ***E-mail address:***

If you agree to participate in this study, please fill in and sign the attached consent form, of which you will receive a copy.

- Supplementary Appendix II –

INFORMED PATIENT CONSENT

Title: EFFICACY OF DIETARY MODULATION WITH SIMBIOTIC CONTENT ON NUTRITIONAL AND METABOLIC STATUS IN SCHIZOPHRENIA SPECTRUM DISORDERS

I, *(name and surname of the participant),* Mr./Mrs ___________________________,

once I have been duly informed by the researcher of all the aspects related to the project,

DECLARE THAT:

- I have read the information sheet I was given.
- I have been able to ask questions about the proposed research project.
- I have received enough information and have answered all my questions about the study.
- I understand that my participation is voluntary.
- I understand that I can withdraw from the study:

  - Whenever I wish.
  - Without having to provide a reasoned justification.
  - Without this having any effect on my health care.

- I understand that, by participating in the study, I consent to the processing, communication and transfer of my personal and health data under the terms set out in the Information Sheet, which has been provided to me.
- I freely agree to participate in this study.

If the participating subject is unable to write and sign, consent is given orally in the presence of the witnes Mr./Mrs____, who signs below.

**Signature of the Subject / Witness.**	**Signature of the researcher and member number.**
**Date:**	**Date:**

# - - Supplementary Appendix III –

INFORMED CONSENT OF THE PATIENT'S LEGAL REPRESENTATIVE

Title: EFFICACY OF DIETARY MODULATION WITH SIMBIOTIC CONTENT ON NUTRITIONAL AND METABOLIC STATUS IN SCHIZOPHRENIA SPECTRUM DISORDERS

I, *(name and surname of the participant),* Mr./Mrs , as LEGAL REPRESENTATIVE of the resident (*name and surname of the resident)* Mr./Mrs, , once I have been duly informed by the responsible researcher of all aspects related to the project,

DECLARE THAT:

- I have read the information sheet I was given.
- I have been able to ask questions about the proposed research project.
- I have received enough information and have answered all my questions about the study.
- I understand that my participation is voluntary.
- I understand that I can withdraw from the study:
    - Whenever I wish.
    - Without having to provide a reasoned justification.
    - Without this having any effect on my health care.
- I understand that, by participating in the study, I consent to the processing, communication and transfer of my personal and health data under the terms set out in the Information Sheet, which has been provided to me.
- I freely agree to participate in this study.

If the participating subject is unable to write and sign, consent is given orally in the presence of the witnes Mr./Mrs____, who signs below.

**Signature of the Subject / Witness.**	**Signature of the researcher and member number.**
**Date:**	**Date:**
